# The clinical role of rTMS in difficult-to-treat depression

**DOI:** 10.1192/j.eurpsy.2024.62

**Published:** 2024-08-27

**Authors:** B. Baune

**Affiliations:** Department of Psychiatry, University of Münster, Münster, Germany

## Abstract

**Abstract:**

Several meta-analyses demonstrated the efficacy of unilateral High-Frequency Left-sided (HFL) repetitive Transcranial Magnetic Stimulation (rTMS) for individuals with Major Depressive Disorder (MDD); however, results are contradictory due to heterogeneity of the included studies. Empirical evidence on the relative efficacy of rTMS treatment compared with standard pharmacotherapy in Treatment-Resistant Depression (TRD) is presented. Random effects models were used to assess the effects of rTMS on response and remission rates. In 19 randomized double-blinded sham-controlled studies were included for quantitative analysis for response (n = 854 patients) and 9 studies for remission (n = 551 patients), the risk ratio (RR) for response and remission are 2.25 and 2.78, respectively for patients after two treatment failures using rTMS as add-on treatment compared to standard pharmacotherapy. The presentation will conclude, that rTMS is significantly more effective than sham rTMS in TRD in response and remission outcomes and may be beneficial as an adjunctive treatment in patients with MDD after two treatment failures. This finding is consistent with previous meta-analyses; however, the effect size was smaller than in the formerly published literature.

**Disclosure of Interest:**

None Declared

